# High susceptibility to collagen-induced arthritis in mice with progesterone receptors selectively inhibited in osteoprogenitor cells

**DOI:** 10.1186/s13075-020-02242-8

**Published:** 2020-07-02

**Authors:** Lixian Liu, Junjing Jia, Min Jiang, Xueping Liu, Chenling Dai, Barton L. Wise, Nancy E. Lane, Wei Yao

**Affiliations:** 1 Department of Internal Medicine, University of California, Davis Medical Center, 4625 2nd Avenue, Sacramento, CA 95817 USA; 2 Faculty of Animal Science and Technology, Yunnan Agricultural University, Kunming, Yunnan People’s Republic of China; 3 Department of Orthopaedic Surgery, UC Davis Medical Center, Sacramento, 95817 USA

**Keywords:** Progesterone receptor, Inflammatory arthritis, Osteoprogenitor cells, Susceptibility, Sex difference

## Abstract

**Background:**

Progesterone receptor (PR) affects immunomodulation, and lack of PR in osteoprogenitor cells primarily affects pathways associated with immunomodulation, especially in males. In this study, we selectively deleted PR from osteoprogenitor cells using Prx1-Cre to evaluate the tissue-specific effects of PR on the pathegenesis of inflammatary arthritis (IA).

**Methods:**

Collagen-induced arthritis (CIA) was used as an IA animal model. Both male and female PR^ΔPrx1^ mice and their wild-type (WT) littermates were immunized with collagen II (CII) emulsified complete Freund’s adjuvant (CFA). Joint erosion, inflammation, and cartilage damage were assessed using a semiquantitative histologic scoring system. Bone volume and erosions in knee and ankle joints were quantitated using microCT and histology.

**Results:**

Bone erosions developed in both paw joints in 37.5% and 41.7% of the WT and PR^ΔPrx1^ female mice and in 45.4 and 83.3% of the WT and PR^ΔPrx1^ male mice, respectively. Also, both joint damage and subchondral bone erosions were significantly more severe in male PRcKO-CIA mice than in male WT-CIA mice. Female PR^ΔPrx1^ mice also developed higher bone loss in the knee joints than the KO-normal or WT-CIA females although with less severity compared to the male mice.

**Conclusions:**

The presence of PR in osteoprogenitor cells decreased the development of collagen-induced arthritis and might help to explain the sex differences observed in human inflammatory arthritis.

## Introduction

Rheumatoid arthritis (RA) is a systemic autoimmune disease that can affect many organ systems, and inflammation of synovial tissue causes activation of inflammatory cytokines that destroy both cartilage and periarticular bone [1–3]. About 3 million Americans suffer from RA, with nearly three times more women than men affected [4–8]. In women, RA most commonly begins between the ages of 30 and 60 years, but in men, RA often begins later in life. The mechanism for this sexual dimorphism in RA is not clear. Most studies of sex-specific factors affecting RA have focused on sex hormones due to the observation that RA activity is reduced in females during pregnancy and that male RA patients generally have a less severe course of disease and better response to therapy [5, 9, 10]. Estrogen is reported to have both pro-inflammatory and anti-inflammatory effects on the immune system while both progesterone and androgen are anti-inflammatory [11–19]. The effects of hormones are primarily regulated through their hormonal receptors. The presence and proportion of hormone receptors in different tissues and cells, including fibroblasts, chondrocytes, and bone cells, may define their roles in the sexually dimorphic pathogenesis of RA [20–27].

### Potential effects of progesterone on arthritis

Progesterone is a sex-related steroid that has been studied extensively for its effects on the reproductive system. Progesterone’s actions are mainly mediated through the progesterone nuclear receptors A and B (PR-A and B), which are ligand-regulated transcription factors [28]. The presence or absence or relative proportion of PR in different tissues may explain the PR’s sexual dimorphic roles in these tissues [29–31]. In contrast to the estrogen receptor, PR’s role may be more important in immunomodulation in female-dominant diseases such as systemic lupus erythematosus, rheumatoid arthritis, and osteoarthritis [32–34]. However, the immunomodulatory role of PR in musculoskeletal tissue is not well understood. PR is expressed by cultured osteoblasts, osteoclasts [35–37], and chondrocytes [38] and is present in vivo in mouse bone [37, 39]. Utilizing genetic fate mapping and immunohistochemistry techniques, we observed PR (esp. PR-B) expression in articular cartilage and in the growth plate as well as in subchondral bone [39]. We also noted that in PR selective deletion in Prx1+ cells, which give rise to both osteoblasts and chondrocytes, the PR^ΔPrx1^ mice had significantly higher trabecular bone mass as compared to their WT littermates [39]. Additionally, conditional PR deletion in the Prx1+ osteoprogenitor cells significantly suppressed immunomodulatory pathways, especially in the males. The disease pathway analyses and RNA-Seq study suggested that rheumatoid arthritis is a potential disease target for PR modulation [34]. Since the lack of PR signaling in the osteoprogenitor cells (OPC) regulated immunomodulation pathways [34], we performed this study to evaluate the role of PR in the PR^ΔPrx1^ mice using a CIA model.

## Methods

### Mice and collagen-induced arthritis (CIA) model

PR-flox mice were obtained from Baylor College of Medicine (Houston, TX, USA). A targeting vector designed to replace part of exon 2 of the PR gene with a selectable marker was employed to create a strain of mice carrying a conditional null PR allele [40]. Prx1-Cre mice were purchased from the Jackson Laboratory. Eight-week-old female and male mice were immunized with 100 μg chicken collagen in completed Freund’s adjuvant (CFA) (Chondrex Inc. Redmond WA USA). On day 21, the mice were boosted with 100 μg chicken collagen in in-completed Freund’s adjuvant (IFA) subcutaneously. On day 24, all mice received 50 μg LPS *E. coli* O111: B4 (Sigma St. Louis, MI USA) via intraperitoneal injection (i.p.) in normal saline. The mice were euthanized on day 50. The onset of the CIA usually occurs on day 26, after initial immunization, and the disease model generally lasts 40 days [41–45].

PCR-based strategies were used for genotyping mouse genomic DNA. All animal work was done in compliance with the guiding principles of UC Davis’s “Care and Use of Animals.” Mice were housed in the animal facility under strictly controlled environmental conditions (12-h light/dark cycle, room temperature 22 °C), and fed ad libitum (food and water). The Institutional Animal Care and Use Committee of the University of California Davis approved the animal protocol.

### T cell stimulation for FACS

Total mononuclear cells were collected from peripheral blood using the Ficoll-Paque density gradient method. The cells were then incubated with phorbol 12-myristate 13-acetate (PMA) in combination with ionomycin for 3 days before running fluorescence-activated cell sorting (FACS). We used the following key markers for activated T cells CD3/PerCP-Cy5.5 (Total T), CD25/PE-CF594, and CD45RO/PE-Cy7 (R &D Systems, Minneapolis, MN, USA).

### Measurements of inflammation, bone erosion, and cartilage damage

Whole knee and ankle joints were fixed, decalcified, embedded in paraffin, and stained with hematoxylin or Safranin-O. Inflammation was scored semi-quantitatively from 0 to 5: 0 = normal; 1 = minimal infiltration of inflammatory cells and/or mild edema; 3 = moderate infiltration; 4 = marked infiltration; and 5 = severe infiltration. For bone erosion, joint sections were stained for tartrate-resistant acid phosphatase (TRAP) and counterstained with hematoxylin (Sigma St Louis, IL, USA). A score of 0–5 was assigned for bone erosion: 0 = normal; 1 = minimal (small areas of bone resorption, not readily apparent on low magnification); 2 = mild (more areas of resorption in trabecular and cortical bone); 3 = moderate (obvious bone resorption of trabecular and cortical bone, without defects in cortex or loss of trabeculae); 4 = marked (full-thickness defects in cortical bone and marked trabecular bone loss); and 5 = severe (defects in the entire cortex, marked trabecular bone loss) [46–48]. Total TRAP+ cells within the subchondral area were counted and presented as TRAP+ cell/bone surface. Cartilage damage was calculated by the loss of Safranin-O staining that was scored on a semi-quantitative scale from 0 to 4: 0 = intact; 1 = minor (< 10%); 2 = moderate (10–50%); 3 = high (50–80%); and 4 = severe (80–100%) [49, 50]. Two blinded observers performed all the scorings. Data are presented as the average of the scores of both observers.

### Bone mass measurements by microCT

The right knee joints including both the distal femurs (DFM) and the proximal tibiae were scanned and analyzed using VivaCT 40 (Scanco Medical, Bassersdorf, Switzerland) with a voxel resolution of 10 μm in all three spatial dimensions and a mono-energetic (70 Kev) X-ray source. We evaluated the entire knee covering a total of 645 mm in length centered around the knee joint to obtain total knee bone volume/tissue volume (BV/TV) ratio [34, 51, 52] using 3D image-registration schemes Gaussian filters of sigma = 0.8, support = 1, and threshold = 180 for total knee and DFM. Gaussian filters of sigma = 1, support = 2, and threshold = 280 were applied to register the paw.

### Knee histopathology

The left knee joints were fixed in 10% phosphate-buffered saline formalin for 2 days, decalcified in 10% EDTA for 3 weeks, and embedded in paraffin. Sections were stained with Safranin-O—Fast green for measurement of articular cartilage thickness, subchondral bone plate thickness, subchondral trabecular bone number and diameter, and cartilage content using Bioquant Imaging software (Bioquant Imaging System, Nashville, VA USA) [51, 52].

### Statistical analysis

The results are expressed as mean ± standard deviation for bone structure measures, bone turnover, and bone strength variables. Two-way ANOVA was used to account for genotype and sex. If significant differences were observed, then a Sidak’s multiple comparisons test was used to assess pairwise comparisons. A value of *p* < 0.05 was considered statistically significant. Data were analyzed using the GraphPad Prism 8 software package (La Jolla, CA, USA).

## Results

### Mice with PR conditionally knocked out in osteoprogenitor cells (OPC) had higher systemic activation of T cells and showed higher incidence of arthritis

**Fig. 1:**
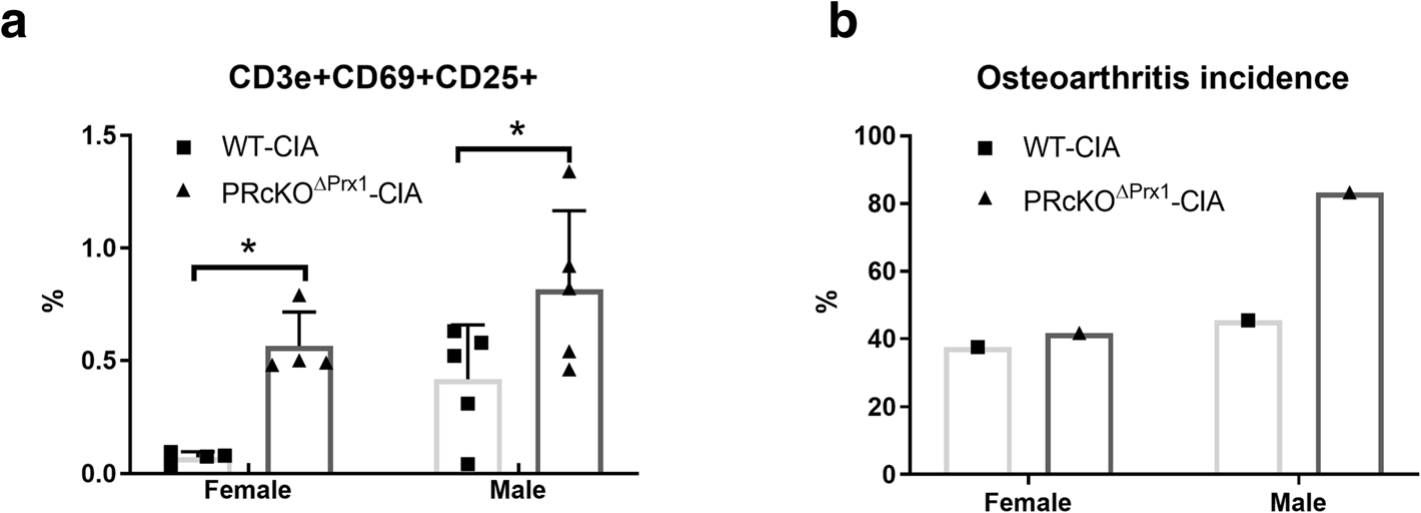
Mice with PR conditionally knocked out in osteoprogenitor cells had higher circulating T cells and higher arthritis incidence in male mice. (**a**) Mononuclear cells were obtained from peripheral blood in WT-CIA or PR^ΔPrx1^-CIA and subjected to FAC determination of CD3e+, CD69+, and CD25+ T-cells. (**b**) The incidence of arthritis was scored using 3D images of paws showing bone erosion at least in one of the paws. *p < 0.05 between indicated groups

We found very low levels of circulating activated T cells marked by CD3e+, CD69+, and CD25+ in the WT-CIA controls, especially in the female WT-CIA mice, at proximately 0.1% of the total mononuclear cells. On the other hand, both female and male PR^ΔPrx1^-CIA mice had significantly higher circulating levels of activated T cells as compared to the WT-CIA mice at day 50 (Fig. 1a). The incidence of arthritis (defined as developing bone erosions viewed by 3D microCT reconstructions of paw images) was 37.5% and 41.7%, respectively, in the female WT and PR^ΔPrx1^-CIA mice and 45.4 and 83.3%, respectively, in the male WT and PR^ΔPrx1^-CIA mice (Fig. 1b). Hence, we observed an increase in systemic inflammation and development of arthritis in the paws in mice lacking PR in the MSCs, highlighting a possible role of PR in systemic as well as local tissue involvement during inflammatory arthritis.

### PR^ΔPrx1^ mice with collagen-induced arthritis had higher levels of bone destruction

**Fig. 2:**
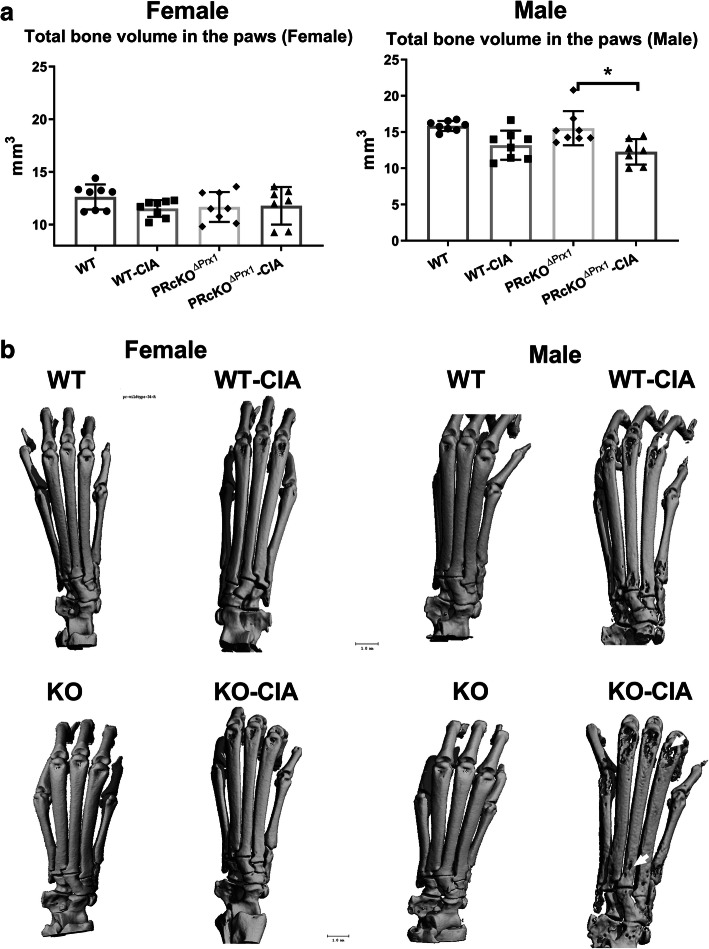
Mice with PR conditionally knocked out in osteoprogenitor cells had a lower bone mass in the paws of male PR^ΔPrx1^-CIA mice. (**a**) The total bone volume of the right paws was measured by microCT in WT and PR^ΔPrx1^ normal or CIA mice. (**b**) Representative microCT paw images from WT or PR^ΔPrx1^ normal or CIA mice. White arrows illustrated bone erosion. *p < 0.05 between indicated groups. Scale bar = 1 mm

**Fig. 3:**
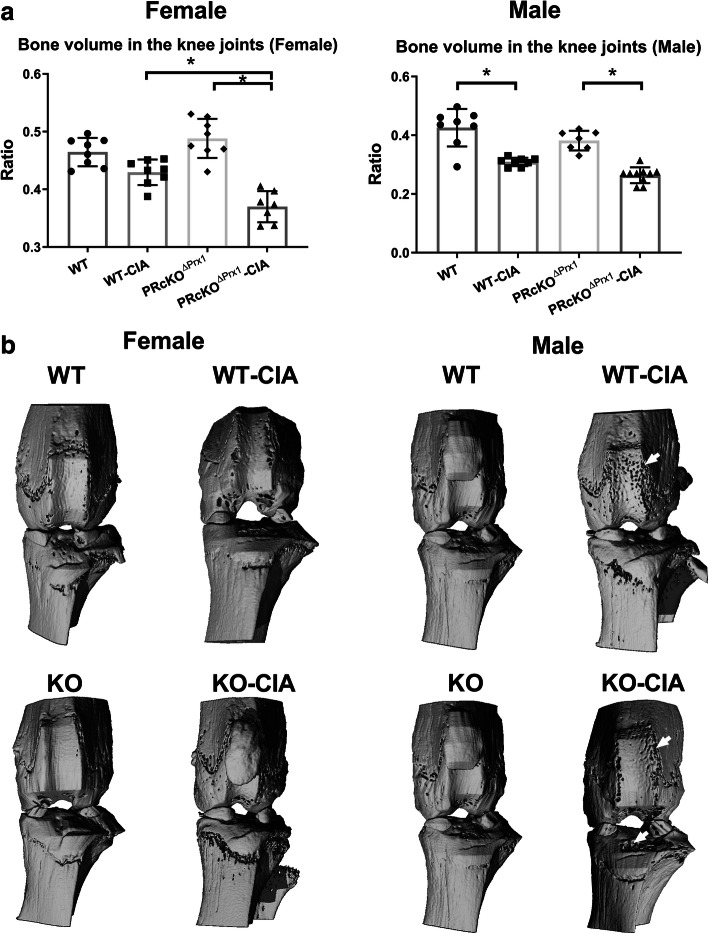
Mice with PR conditionally knocked out in osteoprogenitor cells had a lower bone mass in the knee joints of PR^ΔPrx1^-CIA mice. (**a**) Total bone volume/tissue volume of right knees was measured by microCT in WT and PR^ΔPrx1^ normal or CIA mice. (**b**) Representative microCT knee images from WT or PR^ΔPrx1^ normal or CIA mice. White arrows indicated bone erosions. *p < 0.05 between indicated groups

**Fig. 4:**
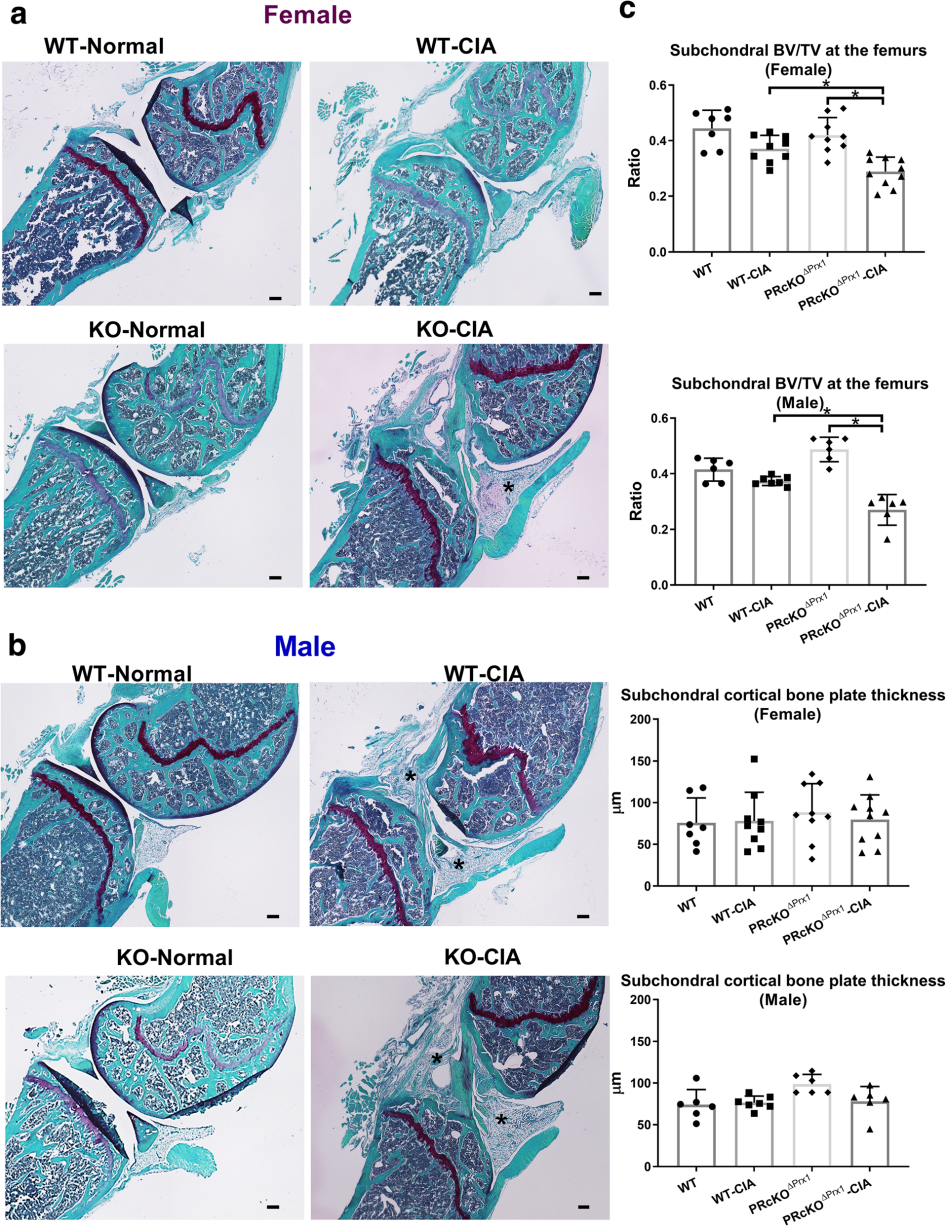
PR^ΔPrx1^-CIA mice had lower subchondral trabecular bone volume. (**a**) Representative Safranin-O-stained knee histologic images from female or (**b**) male in WT or PR^ΔPrx1^ normal or CIA mice. (**c**) Quantitative measurements of subchondral trabecular bone or cortical plate thickness at the femoral epiphyses. Black stars indicate inflamed synovial tissue. *p < 0.05 between indicated groups. Scale bar = 100 μm

**Fig. 5:**
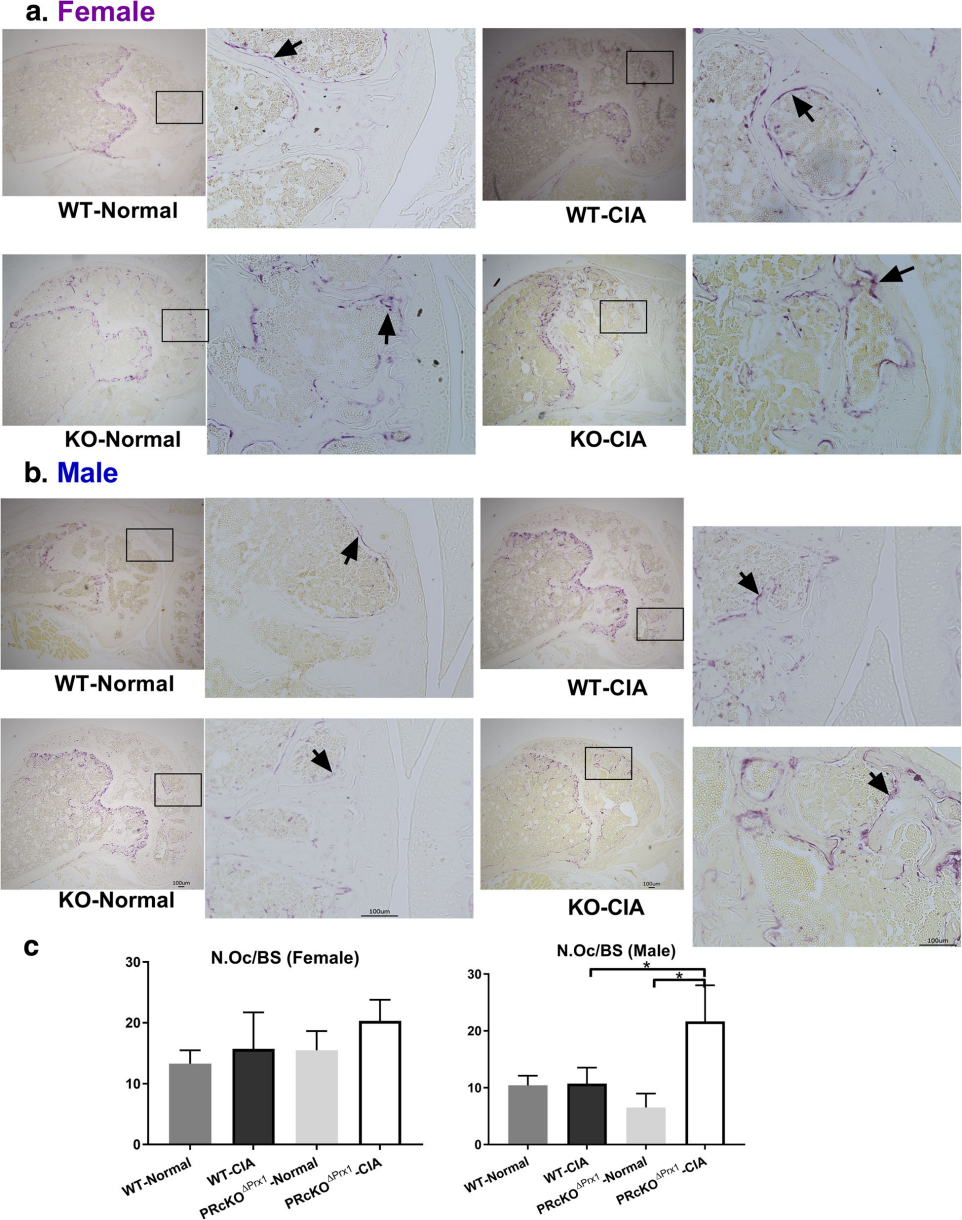
Male PR^ΔPrx1^-CIA mice had higher amounts of surface osteoclasts at the distal femoral subchondral bone. (**a**) Representative TRAP-stained knee histologic images from female or (**b**) male in WT or PR^ΔPrx1^ normal or CIA mice. (**c**) Quantitative measurements of TRAP+ surface osteoclasts at the distal femoral subchondral bone. Black arrows illustrate TRAP+ cells at bone surfaces. *p < 0.05 between indicated groups. Scale bar = 100 μm

MicroCT and histochemical analyses were used to assess the degree of bone erosion in the ankle and the knee joints in WT-CIA and PR^ΔPrx1^-CIA mice and the control mice which did not receive immunizations. In the paws, total bone volume did not differ in WT-CIA mice compared to their WT-non-CIA controls, but was reduced significantly in male PR^ΔPrx1^-CIA mice compared to the PR^ΔPrx1^-non-CIA male controls (Fig. 2a). Areas of bone erosion were present on microCT images of paws, especially in the distal and proximal ends of the metacarpus as well as in the carpus, in the male WT-CIA and PR^ΔPrx1^-CIA mice (Fig. 2b, white arrows). Compared to the non-CIA mice, female PR^ΔPrx1^-CIA, male WT-CIA, and male PR^ΔPrx1^-CIA mice had reduced total bone volume in knee joints. The female PR^ΔPrx1^-CIA had significantly higher bone loss in the knee joint compared to the female WT-CIA mice (Fig. 3a). The non-CIA PR^ΔPrx1^ mice had smooth and continuous bone surfaces in their knees, while focal peri-articular bone erosions were apparent in both the female and male PR^ΔPrx1^-CIA mice (Fig. 3b, white arrows). Histologic measurements confirmed the absence of trabecular bone loss at the femoral subchondral bone in the female and male WT-CIA mice (Fig. 4a–c). Both female and male PR^ΔPrx1^-CIA mice had lower trabecular bone volume compared to their sex-matched PR^ΔPrx1^-non-CIA and WT-CIA mice, with similar subchondral cortical bone plate thickness across all the groups (Fig. 4c). TRAP histochemistry was used to determine the numbers of osteoclasts at the distal femurs, with a focus on the subchondral bone erosions. TRAP+ cell numbers were similar in female and male WT-CIA mice compared to their WT-normal controls. The female PR^ΔPrx1^-CIA mice had a trend of increased TRAP+ cells in the subchondral bone area but did not reach statistical significance when compared to PR^ΔPrx1^-normal control mice. In contrast, more TRAP+ cells were present on the femoral subchondral trabecular bone surface in the male PR^ΔPrx1^-CIA mice compared to male normal and male WT-CIA mice (Fig. 5, black arrows). Taken together, these results suggest that both female and male PR^ΔPrx1^ mice developed more severe bone loss in the knee joints. The male PR^ΔPrx1^ mice are more susceptible to bone loss in the paw and developed higher bone erosion in the knee joints compared to their sex-matched WT-CIA mice.

### Male PR^ΔPrx1^ mice with collagen-induced arthritis had more cartilage damage than WT-CIA control

**Fig. 6:**
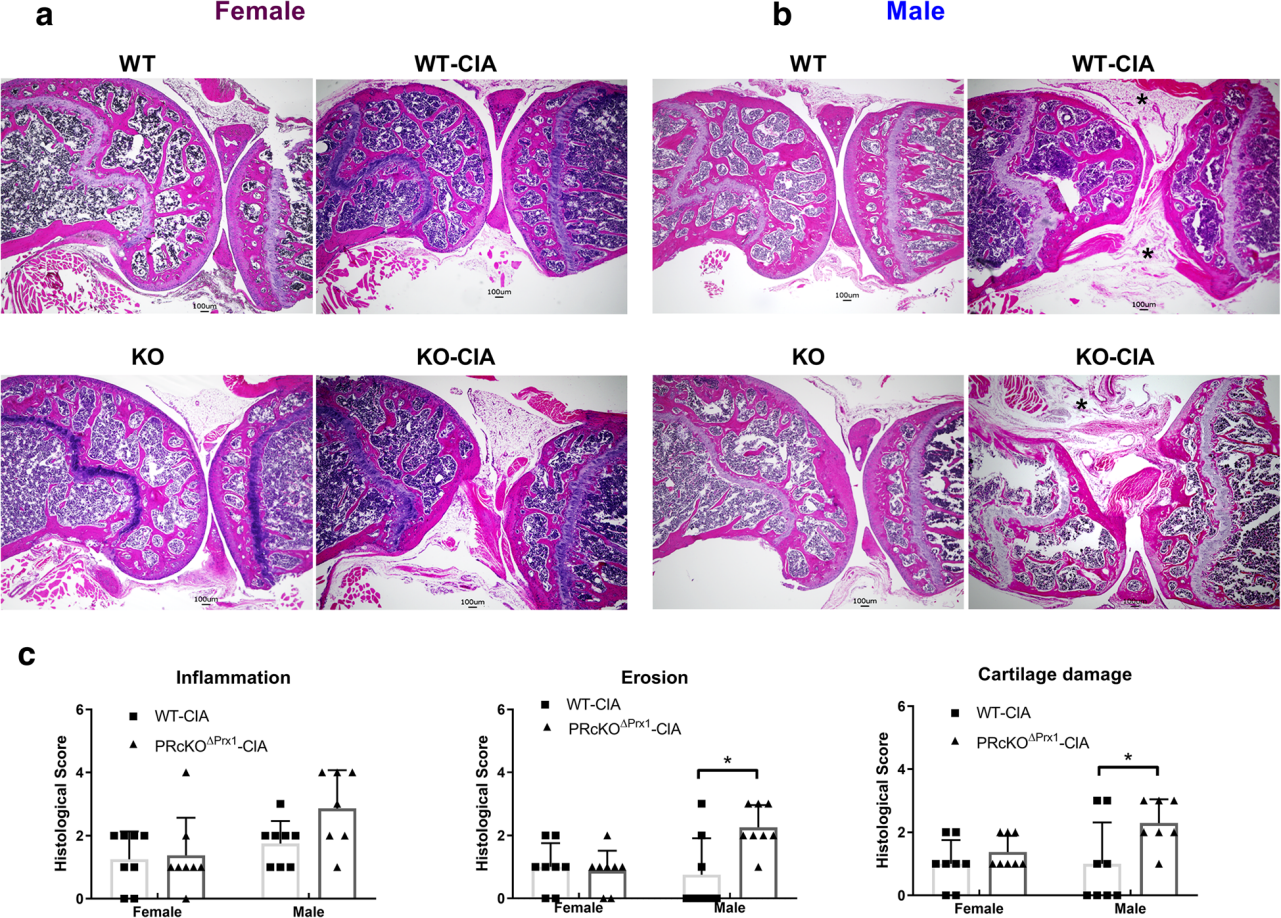
Male PR^ΔPrx1^-CIA mice had more severe knee joint erosion and cartilage damages. (**a**) Representative H&E-stained knee histologic images from female or (**b**) male in WT or PR^ΔPrx1^ normal or CIA mice. (**c**) Semi-quantitation of inflammation, subchondral bone erosion, and cartilage damage of the knee joints. Black stars indicate inflamed synovial tissue. *p < 0.05 between indicated groups. Scale bar = 100 μm

Cartilage destruction and inflammation were assessed on H&E- and Safranin-O-stained sections. The overall semi-quantitative scoring on the H&E-stained sections revealed more inflammation and erosions in the male PR^ΔPrx1^-CIA mice compared to WT-CIA mice (Fig. 6). Examination of Safranin-O-stained knee samples revealed a loss of articular cartilage, especially in the male CIA mice (Fig. 4a, b). In the male PR^ΔPrx1^-CIA mice, there was an almost complete loss of articular cartilage in areas of subchondral bone erosion (Figs. 4 and 6). A similar area of subchondral bone erosion and articular cartilage loss was present in the male WT-CIA as well. Cartilage loss was noted adjacent to inflamed synovium tissues, especially in male WT and PR^ΔPrx1^-CIA mice (Fig. 6a, b). The semi-quantitative erosion and cartilage damage scores were higher in the male PR^ΔPrx1^-CIA mice than in the WT-CIA mice (Fig. 6c).

## Discussion

Mice lacking progesterone receptor signaling in the osteoprogenitor cells were more susceptible to collagen-induced arthritis, especially male mice. The PR^ΔPrx1^-CIA mice, especially the males, had a significantly higher incidence of arthritis, joint inflammation, bone erosion, and cartilage damage compared to the normal male PR^ΔPrx1^ mice or WT-CIA mice. Our findings indicate that under “normal” conditions, the presence of PR in osteoprogenitor cells might be protective against inflammatory arthritis and may also contribute to the sex differences that are observed in RA patients [53–55].

A number of susceptibility genes for RA have been previously identified. The human leukocyte antigen (HLA) is a genetic site controlling immune responses in RA [56, 57]. Several genes outside the HLA region, including *Stat4*, the TRAF1-C5 locus, and PTPN22, have been reported to be associated with activation and progression of inflammation in RA [58–62]. Sex disparities in genetic susceptibility to RA are understudied, and only a polymorphism in the *Cyb5a* gene, which is related to androgen synthesis, has been found to be associated with risk for RA in women but not in men [63]. Recent studies have also suggested a role for epigenetic modifications in the activation and aggressiveness of synovial fibroblasts [64–67] and the X-encoded genes, *Timp1* and *IL-9R* in RA [68]. Some of these epigenetic modifications correlate with X-linked miRNA, and the presence of the second X chromosome in females may affect miRNA expression levels, potentially helping to explain sex-related autoimmunity [69, 70]. Most of these studies on sex-specific factors affecting RA have focused on the potential effects of sex hormones due to the observation that RA improves during pregnancy and that male RA patients generally have a less severe course of illness and better response to therapy [5, 9, 10]. Estrogen has been reported to have both pro-inflammatory and anti-inflammatory effects on the immune system while both progesterone and androgen are anti-inflammatory [11–19]. The effects of hormones are mainly regulated through their hormonal receptors. The presence and proportion of estrogen and androgen receptors in different tissues and cells, including fibroblasts, chondrocytes, and bone cells, might define their roles in the sexually dimorphic pathogenesis of RA [20–27]. We and others have found PR expressed in growth plate chondrocytes, osteoclasts, and osteoblasts, and PR has a critical role in peak bone mass determination in mice [37, 71, 72]. Loss of PR signaling in osteoprogenitor cells regulates key signaling pathways for immune response, especially in males [34]. We identified PR-targeted genes that regulated sex differences, including an “X-inactive specific transcript,” *Xist*, *Mtus2*, *Aldhla7/1*, *Tusc5*, *Cd300c*, and *Pde3a* [34]. The upregulation of Xist is associated with chronic inflammation and pain in females with complex regional pain syndrome [73] and contributes to RA progression [74]. Cd300c and Pde3a are over-presented in RA patients [75, 76] and are associated with inhibition of T cell immunity [77] or response to TNF inhibitors in RA patients [78]. Our prior and current findings [34, 37, 39, 71] suggest that PR may regulate susceptibility to inflammatory arthritis in mice.

The presence of marginal bone erosions, detected by imaging, predicts a more severe disease course with more disability and increased morbidity. The significance of erosions in RA has been the focus of the development and approval of several agents for modifying the course of RA and has been validated in clinical trials as being able to reduce structural joint damage, including bone erosion and cartilage degradation [79, 80]. The bone erosions in RA show a predilection for specific anatomic sites such as the radial aspects of finger joints, while the ulnar aspects are relatively spared [81]. These focal erosions typically emerge at the site at which the synovium comes into direct contact with the bone which is known as bare areas. Anatomical factors that predispose these skeletal sites for erosion include the presence of mineralized cartilage, the insertion of ligaments at the bone surface, and inflamed tendon sheaths that enable the spread of inflammation from the tendon to the articular synovium. Articular erosion at these “bare areas” represents localized bone loss from osteolysis, which resulted from an imbalance in which bone resorption by osteoclasts is predominant over bone formation by osteoblasts. Once established, these bone erosions rarely repair despite the use of potent biologic therapeutic strategies including biologics such as TNF, IL-1, or IL-6 receptor blockade [82–85]. Aberrant repair of erosions appeared as sclerosis with new bone apposition at the base of the erosion and might involve the juxta-articular bone marrow. Adipose tissue might populate the erosive area. Bone erosion seemed to correlate with on-going inflammation. Our study provides additional information to better understand the potential PR regulation of the inflammation-induced bone resorption coupling mechanism in the process of joint and bone damage and how potentiation of this coupling from lack of PR signaling contributes to bone and joint tissue loss in RA in a sex-dependent manner.

RA is a systemic autoimmune disease that induces inflammation of the synovial tissue and causes activation of inflammatory cytokines that destroy both cartilage and peri-articular bone. One of the main shortcomings for the study was the lack of measurements of cytokines and chemokines systemically or locally in the joint tissue. Therefore, we could not directly determine if the PR regulation of joint inflammation and bone loss were directly associated with changes in the cytokine/chemokine levels during the pathogenesis of IA or with the lack of PR expression in the osteoprogenitor cells. Nevertheless, our data suggested that PR might alter the susceptibility to inflammation, cartilage damage, and bone destruction in RA.

## Conclusions

In conclusion, lack of PR in osteoprogenitor cells increased susceptibility to IA, especially in male mice. Our findings indicate that the presence of PR in osteoprogenitor cells decreases the development of collagen-induced arthritis and might also help to explain sex differences observed in rheumatoid arthritis.

## Data Availability

The datasets used and/or analyzed during the current study are available from the corresponding author on reasonable request.
